# Androgen receptor genotypes predict response to endocrine treatment in breast cancer patients

**DOI:** 10.1038/bjc.2011.441

**Published:** 2011-10-27

**Authors:** K B Lundin, M Henningson, M Hietala, C Ingvar, C Rose, H Jernström

**Affiliations:** 1 Department of Oncology, Clinical Sciences, Lund University, Barngatan 2B, Lund SE-22185, Sweden; 2 Department of Surgery, Clinical Sciences, Lund University and Skane University Hospital, Lund, Sweden; 3 Head of the Division of Cancer and Haematology, Skane University Hospital, Sweden

**Keywords:** androgen receptor, breast cancer, oestrogen receptor *α*, genotype, single-nucleotide polymorphism

## Abstract

**Background:**

The androgen receptor (AR) is frequently expressed in breast cancers. The *AR* genotype may affect disease-free survival and response to endocrine therapy.

**Methods:**

In all, 634 women undergoing breast cancer surgery between 2002 and 2008 were followed until 30 June 2010. Six haplotype-tagging single-nucleotide polymorphisms in the *AR*, and the resulting *AR* diplotypes, were examined in relation to breast cancer patient characteristics, tumour characteristics, disease-free survival, and response to endocrine treatment.

**Results:**

Five common *AR* diplotypes were found. Seventeen rare variants were combined into a composite group. The resulting six *AR* diplotype groups were clustered into two subgroups, groups A (*n*=128) and B (*n*=499), with three diplotypes in each. Patients in group B had larger total breast volume (*P*=0.024), higher body mass index (BMI) (*P*=0.050), more axillary lymph node involvement (*P*_trend_=0.020), and higher histological grade (*P*_trend_=0.031). There were 59 breast cancer events in the 569 patients with invasive cancers and no preoperative treatment. Patients in group B also had shorter disease-free survival (*P*=0.037) than patients in group A. Among patients in group B with oestrogen receptor *α* positive tumours, tamoxifen (TAM) treatment was associated with longer disease-free survival (*P*=0.008), while treatment with aromatase inhibitors (AIs) was not (*P*=0.94). Response to endocrine treatment could not be predicted based on BMI, suggesting that the effect of *AR* diplotypes went beyond that of a higher BMI.

**Conclusion:**

A marker for a group of patients who responded to TAM, but not to AIs, was identified. If this finding is confirmed, *AR* genotyping may provide useful information for selection of endocrine treatment of breast cancer patients.

Breast cancer is the most common malignancy among women in Sweden. Over 7000 women are diagnosed with breast cancer every year (The National Board of Health and Welfare; http://www.socialstyrelsen.se). Polymorphisms in genes regulating hormone and growth factor levels have been associated with disease progression and therapeutic outcome in several cancers arising from tissues under hormonal influence (Giwercman *et al*, 2004; Piersma *et al*, 2007; Sissung *et al*, 2011).

Both oestrogens and androgens are important for normal breast development (Dimitrakakis and Bondy, 2009). The balance between the stimulatory effects of oestrogens and the inhibitory effects of androgens functions as a critical factor that regulates mammary cell proliferation, in normal as well as in cancer tissues. Androgens exert their effect in the mammary epithelial cell via two separate pathways, either directly via the androgen receptor (AR), or indirectly through aromatisation to oestrogen. Results from preclinical studies suggest that testosterone may function as a natural, endogenous protector of the breast and limit mitogenic and cancer promoting effects of oestrogens on mammary epithelium (reviewed by Somboonporn and Davis, 2004). However, in postmenopausal women, who have low levels of circulating oestrogens and increased aromatase activity, higher androgen levels have been associated with a small increase in breast cancer risk.

The AR functions as a transcription factor, which regulates the activity of other genes (Gao *et al*, 2005). It is expressed in ∼70–90% of primary breast tumours, closely reflecting the frequency of oestrogen receptor *α* (ER) expression (Birrell *et al*, 2007; Hanley *et al*, 2008; Park *et al*, 2010; Hu *et al*, 2011), and in 75% of breast cancer metastases (Birrell *et al*, 2007). Low AR expression in ER-positive breast cancer has been associated with significantly reduced relapse-free and overall survival (Peters *et al*, 2009).

Endocrine treatment options for patients with ER-positive breast cancers currently include tamoxifen (TAM) and various aromatase inhibitors (AIs) (Bardia and Stearns, 2010; Burstein *et al*, 2010; Colleoni and Giobbie-Hurder, 2010; Hackshaw *et al*, 2011). In randomised trials of unselected patients, AIs were shown to have a better effect than TAM (Baum *et al*, 2003; Burstein *et al*, 2010). Since adjuvant therapy does not work as intended for a considerable number of patients (Early Breast Cancer trialists’ Collaborative Group (EBCTGC), 2005; Colleoni and Giobbie-Hurder, 2010; Dowsett *et al*, 2010), there is a need to identify markers for better selection of endocrine treatment. It has been suggested that response to TAM treatment depends on the *CYP2D6* genotype, although the results are widely heterogeneous (Punglia *et al*, 2008; Lash *et al*, 2009; Dunn *et al*, 2010; Higgins and Stearns, 2011). *CYP2D6* genotyping before TAM treatment is currently not recommended (Lash *et al*, 2009; Burstein *et al*, 2010). Response to AI treatment may depend on the *CYP19A1* genotype (Colomer *et al*, 2008; Fasching *et al*, 2008), but is currently not carried out before selection of endocrine treatment.

The efficacy of TAM has been shown to be equal in obese and non-obese patients (Dignam *et al*, 2003), while a high body mass index (BMI) has been associated with worse response to AI treatment (Pfeiler *et al*, 2011). In women, the density of the AR appears to be higher in visceral than in subcutaneous tissue and the effects of androgens are associated with body fat composition (Bjorntorp, 1997). Androgen receptor overexpression may enhance TAM's agonistic properties in breast cancer and contribute to resistance (De Amicis *et al*, 2010). Conversely, increased androgens and AR expression following AI treatment may contribute to reduced tumour cell proliferation (Chanplakorn *et al*, 2011). Androgen receptor signalling may also be dependent on the AR genotype (Rodriguez-Gonzalez *et al*, 2009). Body constitution may therefore impact on the relationship between androgens, AR and endocrine treatment response.

The present study focussed on a set of six haplotype-tagging single-nucleotide polymorphisms (htSNPs) in the *AR* (Figure 1). These SNPs were previously identified to capture 95% of the haplotypes found in Swedish men. The haplotypes were associated with prostate cancer risk (Lindstrom *et al*, 2006). Since the *AR* gene is located on chromosome X and women carry two copies, we investigated diplotypes rather than haplotypes. To our knowledge, there is only one study published on androgen levels in women in relation to these *AR* diplotypes (Hietala *et al*, 2011). The study reported a weak correlation between diplotype and plasma androgen levels in premenopausal women. The correlation was modulated by exogenous hormone use.

The aims of the present study were to compare the frequency of the *AR* diplotypes in a cohort of women diagnosed with breast cancer with the frequencies found by Hietala *et al* (2011), and to investigate whether any of the *AR* diplotypes were associated with patient characteristics, specifically BMI and waist–hip ratio (WHR), and tumour characteristics. In addition, the study aimed to elucidate whether *AR* diplotypes were associated with breast cancer-free survival independent of treatment, or predicted response to endocrine therapy in patients with ER-positive tumours.

## Subjects and methods

### Breast cancer patients

Women assessed preoperatively at Lund University Hospital in Southern Sweden for a first breast cancer were invited to take part in an ongoing study regarding genetic and non-genetic factors that could be associated with breast cancer prognosis and treatment response. Patients were included between October 2002 and October 2008. Women were invited to participate regardless of ethnic background, age, and stage. The vast majority of women included were ethnic Swedes. Patients with a previous breast cancer or who had been diagnosed and treated for another type of cancer within the past 10 years were not eligible to participate. The Ethics Committee of Lund University approved the study. Written informed consent was obtained from all patients. A total of 634 women were included in the study.

During the preoperative visit, a trained research nurse collected blood samples and measured body weight, height, waist and hip circumferences, and breast volume. ‘Breast volume’ was defined as the sum of the volumes of the right and left breasts. The volume of each breast was measured using plastic cups employed by plastic surgeons doing breast reductions and reconstructions. These cups come in 11 sizes ranging from 200 to 2000 ml as previously described (Ringberg *et al*, 2006).

All patients filled out a preoperative questionnaire including questions on reproductive history, use of exogenous hormones, and concomitant medications during the past week. Follow-up questionnaires were completed at 3–6 months, and 1, 2, 3, 5, and 7 years postoperatively. Information including type of surgery, adjuvant treatment, sentinel node biopsy, and axillary node dissection was obtained from each patient's chart. Tumour size, histological type and grade, axillary node involvement, signs of distant metastases, ER and progesterone receptor (PR) status were obtained from each patient's pathology report. Oestrogen receptor and PR status were determined by immunohistochemistry using the Dako LSAB kit system (Dako) and the antibodies M7047 (ER) and M3569 (PR). Tumours with >10% positive nuclear staining were considered ER positive or PR positive (Bågeman *et al*, 2008). All tumours were analysed at the Department of Pathology of Skåne University Hospital in Lund. Date of death was obtained from the Swedish Population Registry.

Breast cancer surgery is performed at seven different hospitals in the South Swedish Health Care Region, with Skåne University Hospital in Lund serving almost 300 000 inhabitants. Since breast cancer patients are not referred to other hospitals for surgery, this study is population based. According to data obtained from the Regional Tumour Registry, 1139 women with breast cancer were registered in Lund between October 2002 and October 2008, and 1090 received surgical treatment. Six hundred and thirty-four patients (58%) were included in the present study. The majority of the non-participating patients did not decline participation, but were missed due to lack of available research nurses. Approximately 5% of patients were missed due to unverified diagnosis at the time of surgery. The median age at surgery for all operated patients was 60.1 years. Oestrogen receptor status was positive in 84.6% of patients and PR status was positive in 68.1%. The follow-up rates for the breast cancer patients (without preoperative interstitial laser thermotherapy or neo-adjuvant treatment) who were alive and recurrence free at each visit were as follows for the 1-year, 2-year, 3-year, 5-year, and 7-year follow-up visits: 98.5%, 95.1%, 92.4%, 94.6%, and 90.8%, respectively.

### SNP genotyping

Genomic DNA was extracted from 300 ml of peripheral blood using the Wizard genomic DNA purification kit (Promega, Madison, WI, USA). Genotyping of the *AR* htSNPs was performed at Region Skåne Competence Centre, Malmö University Hospital, Malmö, Sweden. The genotyping of *AR* rs1337080 was done according to the manufacturer's protocol with TaqMan assay by allelic discrimination based on real-time PCR on an ABI PRISM 7900 Sequence Detection System (Applied Biosystems, Foster City, CA, USA). Analyses of *AR* rs17302090, rs6152, rs7061037, rs5031002, and rs5964607, as well as CYP19A1 rs4646, were performed on a matrix-assisted laser desorption/ionisation time-of-flight mass spectrometry on a Sequenom MassARRAY platform (Sequenom, San Diego, CA, USA), using iPLEX reagents according to the manufacturers’ protocol. For quality control, over 10% of the samples were run in duplicate. The concordance was 100% for the validated samples. The call rates varied between 96.2% and 100%.

### *AR* diplotype construction

Each SNP was cross-tabulated against the other five SNPs. This procedure showed that certain combinations did not exist or were very rare. We therefore constructed the haplotypes and diplotypes based on the most likely combinations. Diplotype variants present in <1% of the patients were classified as rare variants and combined into a single group termed ‘rare diplotypes’. Rs17302090 analysis failed for one patient. Based on the other SNPs, this SNP could be imputed. In 24 patients, results for rs6152 were missing, of which 23 could be imputed based on the results of rs7061037, since the R2 between these two SNPs was 0.963. One of these resulted in two copies of the common minor haplotypes, thus resulting in a rare diplotype, while the other 22 were all common variants. For the remaining subject, the diplotype could not be determined, although it could be assigned to the rare-diplotype group, since all possible variants at this position resulted in a rare diplotype. For rs1337080, analysis failed for 21 patients. For 20 patients, the diplotype could be imputed. The remaining subject could be assigned to the rare-diplotype group. Two of the patients with missing results for rs1337080 also had missing results for rs6152 but could be imputed for both positions, thus resulting in two common diplotypes. SNP analysis failed for rs5031002 in two patients, but the diplotypes could be imputed. Ten patients had missing results for rs5964607, of which two were imputed and one was assigned to the rare-diplotype group. For the remaining seven patients, the SNP data could not be imputed. Thus, a total of four subjects were included in the rare-diplotype group, although complete diplotypes were missing for three of them.

### Data analysis

Statistical analyses were performed using the statistical software SPSS Statistics 19 (IBM, Chicago, IL, USA). Body mass index, WHR, and total breast volume were not normally distributed and were transformed using the natural logarithm (ln). Age, BMI, and WHR were also dichotomised according to the following: age <50 *vs* ⩾50 years, BMI <25 *vs* ⩾25, and WHR ⩽0.85 *vs* >0.85. *χ*^2^ was calculated for dichotomised variables. We also examined whether any of the *AR* diplotypes clustered together with respect to BMI, WHR, and disease-free survival. Mann–Whitney *U*-test was used for comparison of non-parametric variables.

Breast cancer-free survival was calculated from inclusion to diagnosis of a breast cancer event, the last study follow-up, or death due to a non-breast cancer-related cause, whichever came first, before 1 July 2010. A breast cancer event was defined as local or regional recurrence, new breast cancer, or distant metastasis. Patients who had received preoperative treatment (*n*=41, plus one patient with missing information regarding interstitial laser thermotherapy), patients with *in situ* carcinoma (*n*=14), and patients diagnosed with a breast cancer event within 3 months from inclusion (*n*=3) were excluded from the survival analyses. One patient with early metastatic spread had also received preoperative treatment (Figure 2). Kaplan–Meier was used to calculate disease-free survival. Cox regression was used to obtain adjusted hazard ratios (HR), adjusting for age (continuous), axillary node involvement (yes/no), tumour size (pT2+: yes/no), grade (grade III: yes/no), TAM treatment (yes/no), and AI treatment (yes/no). Adjuvant treatment reported in the chart after last follow-up or breast cancer event was not considered. Since this was a hypothesis driven exploratory study, no adjustments for multiple testing were performed (Bender and Lange, 2001). Nominal *P*-values are presented. All *P*-values were two-tailed and regarded as significant at *P*<0.05.

## Results

### *AR* diplotypes

**Figure 1:**
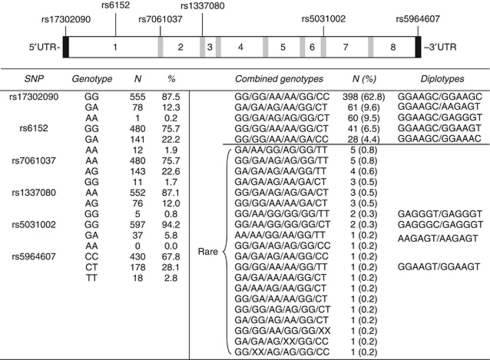
Frequencies of *AR* SNPs and diplotypes among 627 women diagnosed with breast cancer. Genotypes and frequencies are presented for each SNP. Diplotypes present in <1% of the patients were clustered together into a composite group of rare diplotypes. Seven patients were missing due to failed SNP analysis.

*AR* htSNP analyses were performed on 634 patients. A total of 22 different complete genotypes were found in the cohort (*n*=627). The genotype was missing for seven women (Figure 1). An additional three patients had incomplete genotypes, but could be identified as carriers of rare genotypes. Five common diplotype variants were found. Almost 63% of the women were homozygous carriers of the wild-type variant GGAAGC. The other four common diplotypes were all composed of one wild-type allele and one minor allele. These were present in 4.4–9.6% of the women. Seventeen different rare diplotypes were found among 36 patients and were combined into a composite group termed rare diplotypes. An additional three patients had rare diplotypes, although the exact genotype was unknown.

### Patient characteristics

The study included 634 female breast cancer patients, ranging in age from 25 to 99 years, with a median age of 59.6 years. Preoperative patient characteristics are presented in Table 1, and did not differ significantly between the *AR* htSNPs or between the six *AR* diplotype groups when the most common GGAAGC/GGAAGC variant was used as a reference. With respect to BMI, the six diplotypes appeared to cluster in two groups. As shown in Table 1, patients with one of the three diplotypes GGAAGC/AAGAGT, GGAAGC/GGAAAC, or rare diplotypes had a lower median BMI of 23.83 kg m^–2^ (group A) compared with patients with the remaining diplotypes GGAAGC/GGAAGC, GGAAGC/GAGGGT, or GGAAGC/GGAAGT (group B) where the median BMI was 24.77 kg m^–2^ (*P*=0.050). There were no significant differences in WHR. Group B also had larger total breast volume (*P*=0.024).

### Tumour characteristics

**Figure 2:**
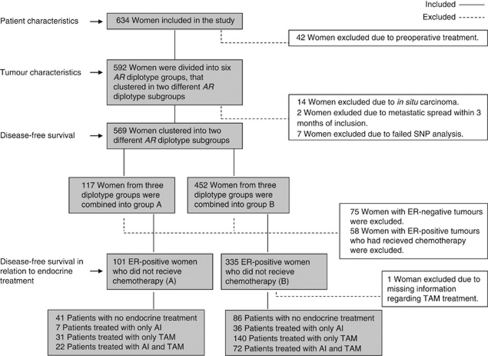
Flowchart displaying patient selection for the different analyses.

The tumour characteristics of the 592 patients who did not receive neo-adjuvant therapy or preoperative interstitial laser thermotherapy (Figure 2) are presented in Table 2. Individual *AR* htSNPs were not significantly associated with tumour characteristics. When comparing the different diplotypes, a trend towards less axillary node involvement (0 *vs* 1–3 *vs* 4+) was seen for the GGAAGC/AAGAGT diplotype, compared with all other diplotypes (*P*_trend_=0.037). Group A had less axillary node involvement (*P*_trend_=0.020) and lower histological grade (*P*_trend_=0.031) compared with group B.

A total of 107 patients had received postoperative chemotherapy. No significant differences in any of the treatment distributions were seen between patients carrying the different *AR* diplotypes.

### Breast cancer-free survival and *AR* diplotypes

**Figure 3:**
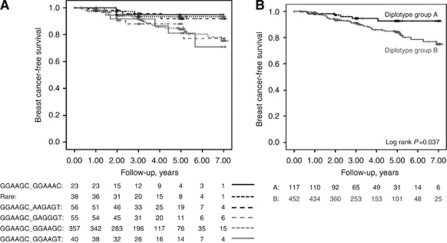
(**A**) Kaplan–Meier survival analysis of disease-free survival with respect to *AR* diplotypes. (**B**) Disease-free survival in group A and group B. Group A includes patients with *AR* diplotypes GGAAGC/AAGAGT, GGAAGC/GGAAAC, or rare diplotypes. Group B includes patients with *AR* diplotypes GGAAGC/GGAAGC, GGAAGC/GAGGGT, or GGAAGC/GGAAGT.

After exclusion of breast cancer events detected on the postoperative metastasis screen, 59 breast cancer events were reported, of which 38 were distant metastases. Breast cancer-free survival in relation to *AR* diplotypes was thus analysed in the 569 patients with invasive cancers and without preoperative treatment (Figure 2). The median total follow-up time was 3.03 years (interquartile range 2.01–4.97). Three diplotypes were associated with longer disease-free survival, while the other three, including the homozygous wild-type variant, were associated with shorter disease-free survival (Figure 3A). Overall, there was no significant difference in disease-free survival between the six diplotype groups. However, the three diplotypes with longer disease-free survival were the same as those in group A. The three diplotypes with shorter disease-free survival were the same as those in group B. Group B was associated with a statistically significantly shorter disease-free survival compared with group A (Log rank *P*=0.037) (HR: 2.38, 95% CI: 1.02–5.55) (Figure 3B). The results became slightly weaker after stratification according to BMI ⩾25 (*P*=0.056). However, group B had shorter disease-free survival in both strata of BMI. Stratification according to BMI ⩾25 alone did not yield any significant differences with respect to disease-free survival. The results remained essentially the same when stratifying according to previous use of hormone therapy.

Among these 569 patients with invasive cancers and no preoperative treatment, group B also presented with a larger total breast volume (*P*=0.007), a higher BMI (*P*=0.011), more axillary lymph node involvement (*P*=0.045), and larger tumours (*P*=0.049) compared with group A. No significant differences in WHR or in endocrine treatment duration were found between groups A and B.

After adjusting for age, tumour characteristics, TAM, and AI treatment, the difference in breast cancer-free survival between groups A and B was no longer statistically significant, although the HR remained approximately the same (adjusted HR: 2.22, 95% CI: 0.95–5.21; *P*=0.065). The results remained essentially the same when using the last disease-free follow-up date or date at diagnosis of a breast cancer event as end points in the follow-up.

### *AR* diplotype groups and endocrine treatment response

Disease-free survival in relation to *AR* diplotype groups and endocrine treatment was then estimated in patients with ER-positive tumours who had not received chemotherapy (*n*=436) (Figure 2). In patients belonging to group A, neither treatment with TAM nor treatment with AI seemed to have any significant effect on breast cancer-free survival. There were only four breast cancer events in group A -- one in a patient with no endocrine therapy, three in patients who had received TAM, and none in the patients who had received AI with or without TAM.

**Figure 4:**
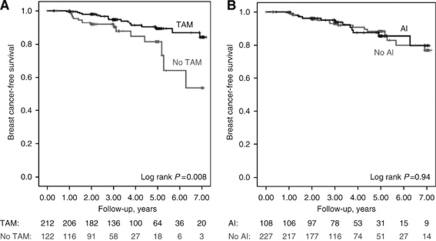
Disease-free survival in group B patients who had ER-positive tumours and who had not received chemotherapy. (**A**) Patients treated with TAM. (**B**) Patients treated with AI.

Patients treated with TAM in group B had significantly longer breast cancer-free survival compared with patients who had not received TAM (Log rank *P*=0.008) (HR: 0.40, 95% CI: 0.20–0.81) (Figure 4A). In contrast, treatment with AI seemed to have no effect on breast cancer-free survival (Log rank *P*=0.94) (Figure 4B). Since some patients had received both TAM and AI while others had received either monotherapy or no endocrine treatment, we first excluded patients who had not received any endocrine therapy. Tamoxifen treatment was still significantly associated with longer disease-free survival (Log rank *P*=0.012), while AI treatment was not. We then excluded patients who had received both TAM and AI. Once again TAM treatment was significantly associated with longer disease-free survival (Log rank *P*=0.036), while AI treatment was not. The results for both TAM and AI were essentially the same after stratifications or adjustments for axillary node involvement, tumour size and BMI. A multivariate model including age, BMI, tumour size, axillary node involvement, grade, as well as both AI and TAM showed that TAM was significantly associated with longer disease-free survival (adjusted HR: 0.29, 95% CI: 0.14–0.64; *P*=0.002), but AI was not (*P*=0.27).

Since AI response may be dependent on the *CYP19A1* SNP rs4646 (Colomer *et al*, 2008; Darabi *et al*, 2011), the rs4646 allele distribution was assessed. The rs4646 distribution did not significantly differ between groups A and B (results not shown). Further adjustment for rs4646 in the multivariate model did not alter the results.

Similar results were obtained when using the last disease-free follow-up date or date at diagnosis of a breast cancer event as end points in the total follow-up, or after inclusion of patients who had received neo-adjuvant therapy or adjuvant chemotherapy.

## Discussion

The main finding of the present study was that adjuvant therapy with TAM, but not with AI, was significantly associated with longer breast cancer-free survival in patients carrying certain *AR* diplotype variants (group B) compared with patients not treated with TAM.

In this study, *AR* diplotypes were analysed in relation to patient and tumour characteristics, endocrine treatment, and disease-free survival. The study included 58% of all breast cancer patients who had received surgery during the same time period. The patients included in the study were comparable to all operated patients with respect to age, ER and PR status as reported by the Regional Tumour Registry. The frequencies of the five most common *AR* diplotypes were also similar to those found in young Swedish women from high-risk breast cancer families (Hietala *et al*, 2011). No significant differences in patient or tumour characteristics were seen between the different *AR* htSNPs or the *AR* diplotypes, except for the GGAAGC/AAGAGT diplotype, which was associated with less axillary node involvement.

The three *AR* diplotypes in group B were associated with shorter disease-free survival compared with the *AR* diplotypes in group A. Patients in group B also presented with higher BMI, larger total breast volume, larger tumours, and a higher frequency of axillary lymph node involvement. However, none of these variables alone could explain the shorter disease-free survival in group B. Moreover, WHR did not differ between groups A and B. Thus, android type obesity or overweight could not explain the shorter disease-free survival in group B (Qiao and Nyamdorj, 2010).

In patients belonging to group B, adjuvant therapy with TAM was significantly associated with longer breast cancer-free survival, compared with patients not treated with TAM. In contrast, adjuvant AI treatment had no effect on breast cancer-free survival in this group of patients, a finding suggestive of AI resistance. Therefore, we analysed the allele distribution of the *CYP19A1* rs4646, which has been suggested to be involved in AI resistance (Colomer *et al*, 2008; Darabi *et al*, 2011). The difference in AI and TAM treatment response in group B was independent of the *CYP19A1* rs4646 variant.

Among patients in group A, neither TAM nor AI treatment had any significant effect on the breast cancer-free survival. However, none of the four breast cancer events in this group was found among the 29 patients who had received AI treatment. This might indicate a better effect on breast cancer-free survival for AI in this *AR* diplotype group, but needs to be confirmed in a larger, independent cohort with longer follow-up.

The mechanisms behind the results remain to be elucidated. The *AR* genotype is associated with AR signalling (Rodriguez-Gonzalez *et al*, 2009). However, the *AR* diplotypes did not tag for CAG or GGC repeat length polymorphisms in the previous study (Hietala *et al*, 2011). Others have shown that the AR is a direct repressor of ER*α* signalling in breast cancer cells (Panet-Raymond *et al*, 2000; Peters *et al*, 2009) due to an association between AR and the oestrogen response elements (Peters *et al*, 2009). It is possible that the *AR* variants constituting group B lead to a reduced ability of the AR to repress the ER*α* signalling. The ER is blocked by TAM, thereby inhibiting oestrogen-dependent cell proliferation. Treatment with AI, on the other hand, seemed to have no effect on the disease-free survival in group B. Aromatase activity is found in adipose tissues, including the breast, and breast tumour tissue expresses aromatase activity (Miller, 2006). In postmenopausal women, aromatase levels are known to increase with increasing BMI, and it has been suggested that the levels reached may exceed the amount that can be successfully inhibited by AIs (Goodwin and Pritchard, 2010). In the present study, patients in group B had higher BMI and larger total breast volume compared with group A, and therefore potentially higher aromatase levels, whereby AI treatment may be less effective. However, endocrine treatment response could not be predicted based on BMI, thus suggesting that the effect of *AR* diplotypes went beyond that of a high BMI.

AIB1 (amplified in breast cancer-1) is a known cofactor for both AR and ER*α* (Gojis *et al*, 2010; Zhou *et al*, 2010). AIB1 expression is frequently increased in breast cancer tissue and has been associated with markers of more aggressive disease. Several *AR* mutations found in prostate cancer tissue have been found to increase the binding affinity to AIB1 (Zhou *et al*, 2010). Moreover, high levels of AIB1 were associated with better response to the AI examestane (Yamashita *et al*, 2009). Thus, ARs in group B may have altered binding affinity for AIB1, possibly affecting the response to AI treatment.

AR expression analysis was not included in the routine analyses, and we were therefore unable to compare *AR* diplotype data with AR expression levels in the tumour. Likewise, information regarding HER-2/neu status and Ki-67 expression was lacking for the majority of patients included in the study. HER-2/neu status has been routinely analysed as of November 2005 and Ki-67 expression as of March 2009. A recent study showed that AR expression was associated with molecular subtypes of breast cancer (Collins *et al*, 2011) and was most frequent in luminal A and B. As the breast tumours were not routinely classified according to molecular subtypes in Lund, we were unable to investigate whether the *AR* diplotypes were associated with any of the molecular subtypes.

Since this was an exploratory study, we presented nominal *P*-values without adjustment for multiple testing (Bender and Lange, 2001). In the present exploratory setting, we feel that Bonferroni correction is too stringent and decreases power. Since there is a risk for false positive findings, the results require confirmation in an independent patient population.

In conclusion, we found a marker for a group of patients who responded to TAM but not to AI treatment. These results warrant confirmation in an independent cohort, preferably with patients who have been randomised to different treatment arms. If confirmed, *AR* diplotype profiling of patients may be useful for selection of endocrine therapy.

## Figures and Tables

**Table 1:** Background characteristics for the whole cohort and the six different *AR* diplotype groups

	**All, *n*=634**		**GGAAGC/GGAAGC (B), *n*=398**	**GGAAGC/AAGAGT (A), *n*=61**	**GGAAGC/GAGGGT (B), *n*=60**	**GGAAGC/GGAAGT (B), *n*=41**	**GGAAGC/GGAAAC (A), *n*=28**	**Rare diplotypes (A), *n*=39**
	**Median (IQR) or %**	***n***	**Median (IQR) or %**	**Median (IQR) or %**	**Median (IQR) or %**	**Median (IQR) or %**	**Median (IQR) or %**	**Median (IQR) or %**
Age at diagnosis, years	59.6 (51.1–66.1)	634	59.7 (50.4–66.5)	58.7 (52.8–67.5)	60.6 (51.8–66.3)	59.6 (53.3–65.3)	59.4 (52.1–64.7)	58.1 (51.3–63.0)
Weight, kg	68.0 (61.0–76.2)	632	68.0 (60.1–78.5)	65.0 (59.0–79.4)	69.2 (65.0–75.8)	68.6 (63.0–76.7)	69.5 (59.3–75.0)	66.0 (58.0–73.0)
Height, m	1.66 (1.62–1.70)	633	1.66 (1.61–1.70)	1.65 (1.62–1.69)	1.67 (1.61–1.70)	1.67 (1.62–1.70)	1.65 (1.60–1.72)	1.65 (1.63–1.68)
BMI, kg m^–2^	24.6 (22.3–27.8)	631	24.8 (22.4–28.0)	23.9 (21.1–28.3)	24.8 (22.8–28.9)	24.8 (22.6–27.7)	23.9 (21.5–27.5)	23.2 (21.7–26.7)
Waist–hip ratio	0.84 (0.78–0.89)	627	0.84 (0.79–0.89)	0.84 (0.78–0.89)	0.84 (0.79–0.92)	0.83 (0.76–0.87)	0.84 (0.78–0.87)	0.83 (0.79–0.86)
Total breast volume^a^, ml	1000 (625–1450)	546	1000 (625–1600)	900 (700–1300)	1025 (700–1500)	825 (600–1325)	700 (500–1250)	650 (500–1300)
Age at menarche, years	13.0 (12.0–14.0)	629	13.0 (12.0–14.0)	13.0 (13.0–14.0)	13.0 (12.0–14.0)	13.0 (12.0–14.0)	13.0 (13.0–14.0)	13.0 (12.0–14.0)
Parous, %	84.7	634	83.4	90.2	91.7	92.7	78.6	76.9
Age at first full term pregnancy, years	25.0 (22.0–28.0)	534	25.0 (22.0–28.0)	25.0 (21.0–29.3)	25.0 (22.0–27.0)	25.0 (22.0–28.0)	24.5 (22.8–29.3)	24.0 (22.0–27.0)
Ever use of hormone replacement therapy, %	45.3	633	44.0	36.0	47.0	56.0	43.0	56.0

Abbreviations: AR=androgen receptor; IQR=interquartile range.

a Eighty breast cancer patients were excluded due to previous breast surgery.

Data are presented as medians with IQRs or as frequencies.

**Table 2:** Tumour characteristics for patients who did not receive neo-adjuvant therapy (*n*=30) or preoperative interstitial laser thermotherapy (*n*=11, and missing information for one patient) before the surgery

	**All, *n*=634, *n* (%)**	**GGAAGC/GGAAGC (B), *n*=398, *n* (%)**	**GGAAGC/AAGAGT (A), *n*=61, *n* (%)**	**GGAAGC/GAGGGT (B), *n*=60, *n* (%)**	**GGAAGC/GGAAGT (B), *n*=41, *n* (%)**	**GGAAGC/GGAAAC (A), *n*=28, *n* (%)**	**Rare diplotypes (A), *n*=39, *n* (%)**
Neo-adjuvant therapy	30 (4.7)	21 (5.3)	3 (4.9)	3 (5.0)	1 (2.4)	2 (7.1)	0
Preoperative interstitial laser thermotherapy	11^a^ (1.7)	7^a^ (1.8)	0	2 (3.3)	0	2 (7.1)	0
No preoperative treatment	*n*=592	*n*=369	*n*=58	*n*=55	*n*=40	*n*=24	*n*=39
*pT*							
*In situ*	14 (2.4)	10 (2.7)	2 (3.4)	0	0	1 (4.2)	1 (2.6)
*1*	424 (71.6)	261 (70.7)	45 (77.6)	34 (61.8)	30 (75.0)	17 (70.8)	31 (79.5)
*2*	144 (24.3)	91 (24.7)	11 (19.0)	20 (36.4)	9 (22.5)	6 (25.0)	6 (15.4)
*3*	9 (1.5)	6 (1.6)	0	1 (1.8)	1 (2.5)	0	1 (2.6)
*4*	1 (0.2)	1 (0.3)	0	0	0	0	0
*Missing*	0	0	0	0	0	0	0
*Histological grade*							
*I*	157 (26.6)	86 (23.4)	25 (43.1)	15 (27.3)	7 (17.5)	9 (37.5)	13 (33.3)
*II*	308 (52.1)	204 (55.4)	22 (37.9)	30 (54.5)	23 (57.5)	9 (37.5)	17 (43.6)
*III*	126 (21.3)	78 (21.2)	11 (19.0)	10 (18.2)	10 (25.0)	6 (25.0)	9 (23.1)
*Missing*	1	1	0	0	0	0	0
*Hormone receptor status*							
*ER+*	502 (86.7)	304 (84.4)	53 (94.6)	46 (85.2)	37 (92.5)	22 (95.7)	33 (84.6)
*ER*−	77 (13.3)	56 (15.6)	3 (5.4)	8 (14.8)	3 (7.5)	1 (4.3)	6 (15.4)
*PR+*	402 (69.4)	255 (70.8)	39 (69.6)	33 (61.1)	28 (70.0)	13 (56.5)	28 (71.8)
*PR*−	177 (30.6)	105 (29.2)	17 (30.4)	21 (38.9)	12 (30.0)	10 (43.5)	11 (28.2)
*Missing*	13	9	2	1	0	1	0
*Axillary node involvement*							
*0*	368 (62.4)	227 (61.9)	42 (72.4)	27 (49.1)	26 (65.0)	15 (62.5)	26 (66.7)
*1–3*	167 (28.3)	101 (27.5)	15 (25.9)	19 (34.5)	11 (27.5)	8 (33.3)	11 (28.2)
*4+*	55 (9.3)	39 (10.6)	1 (1.7)	9 (16.4)	3 (7.5)	1 (4.2)	2 (5.1)
*Missing*	2	2	0	0	0	0	0

Abbreviations: ER=oestrogen receptor; PR=progesterone receptor.

a Information about preoperative interstitial laser thermotherapy was missing for one patient.

Data are presented for all patients (*n*=592) and for the six different *AR* diplotype groups (*n*=585, diplotype information missing for seven patients).

## References

[bib1] Bågeman E, Ingvar C, Rose C, Jernström H (2008) Coffee consumption and CYP1A2^*^1F genotype modify age at breast cancer diagnosis and estrogen receptor status. Cancer Epidemiol Biomarkers Prev 17: 895–901 18398030 10.1158/1055-9965.EPI-07-0555

[bib2] Bardia A, Stearns V (2010) Personal breast: customizing agents and biomarkers for optimal adjuvant endocrine therapy. Breast Cancer Res Treat 120: 437–439 20162380 10.1007/s10549-010-0787-1

[bib3] Baum M, Buzdar A, Cuzick J, Forbes J, Houghton J, Howell A, Sahmoud T (2003) Anastrozole alone or in combination with tamoxifen *vs* tamoxifen alone for adjuvant treatment of postmenopausal women with early-stage breast cancer: results of the ATAC (Arimidex, tamoxifen alone or in combination) trial efficacy and safety update analyses. Cancer 98: 1802–1810 14584060 10.1002/cncr.11745

[bib4] Bender R, Lange S (2001) Adjusting for multiple testing – when and how? J Clin Epidemiol 54: 343–349 11297884 10.1016/s0895-4356(00)00314-0

[bib5] Birrell SN, Butler LM, Harris JM, Buchanan G, Tilley WD (2007) Disruption of androgen receptor signaling by synthetic progestins may increase risk of developing breast cancer. FASEB J 21: 2285–2293 17413000 10.1096/fj.06-7518com

[bib6] Bjorntorp P (1997) Hormonal control of regional fat distribution. Hum Reprod 12(Suppl 1): 21–25 10.1093/humrep/12.suppl_1.21 9403318

[bib7] Burstein HJ, Prestrud AA, Seidenfeld J, Anderson H, Buchholz TA, Davidson NE, Gelmon KE, Giordano SH, Hudis CA, Malin J, Mamounas EP, Rowden D, Solky AJ, Sowers MR, Stearns V, Winer EP, Somerfield MR, Griggs JJ (2010) American Society of Clinical Oncology clinical practice guideline: update on adjuvant endocrine therapy for women with hormone receptor-positive breast cancer. J Clin Oncol 28: 3784–3796 20625130 10.1200/JCO.2009.26.3756 PMC5569672

[bib8] Chanplakorn N, Chanplakorn P, Suzuki T, Ono K, Wang L, Chan MS, Wing L, Yiu CC, Chow LW, Sasano H (2011) Increased 5alpha-reductase type 2 expression in human breast carcinoma following aromatase inhibitor therapy: the correlation with decreased tumor cell proliferation. Horm Cancer 2: 73–81 21761341 10.1007/s12672-010-0062-2 PMC10357990

[bib9] Colleoni M, Giobbie-Hurder A (2010) Benefits and adverse effects of endocrine therapy. Ann Oncol 21(Suppl 7): vii107–vii111 20943602 10.1093/annonc/mdq281 PMC3146255

[bib10] Collins LC, Cole KS, Marotti JD, Hu R, Schnitt SJ, Tamimi RM (2011) Androgen receptor expression in breast cancer in relation to molecular phenotype: results from the Nurses’ Health Study. Mod Pathol 24: 924–931 21552212 10.1038/modpathol.2011.54 PMC3128675

[bib11] Colomer R, Monzo M, Tusquets I, Rifa J, Baena JM, Barnadas A, Calvo L, Carabantes F, Crespo C, Munoz M, Llombart A, Plazaola A, Artells R, Gilabert M, Lloveras B, Alba E (2008) A single-nucleotide polymorphism in the aromatase gene is associated with the efficacy of the aromatase inhibitor letrozole in advanced breast carcinoma. Clin Cancer Res 14: 811–816 18245543 10.1158/1078-0432.CCR-07-1923

[bib12] Darabi H, Czene K, Wedren S, Li Y, Liu J, Hall P, Humphreys K (2011) Genetic variation in the androgen estrogen conversion pathway in relation to breast cancer prognosticators. Breast Cancer Res Treat 127: 503–509 20960227 10.1007/s10549-010-1218-z

[bib13] De Amicis F, Thirugnansampanthan J, Cui Y, Selever J, Beyer A, Parra I, Weigel NL, Herynk MH, Tsimelzon A, Lewis MT, Chamness GC, Hilsenbeck SG, Ando S, Fuqua SA (2010) Androgen receptor overexpression induces tamoxifen resistance in human breast cancer cells. Breast Cancer Res Treat 121: 1–11 19533338 10.1007/s10549-009-0436-8 PMC2995248

[bib14] Dignam JJ, Wieand K, Johnson KA, Fisher B, Xu L, Mamounas EP (2003) Obesity, tamoxifen use, and outcomes in women with estrogen receptor-positive early-stage breast cancer. J Natl Cancer Inst 95: 1467–1476 14519753 10.1093/jnci/djg060 PMC4676737

[bib15] Dimitrakakis C, Bondy C (2009) Androgens and the breast. Breast Cancer Res 11: 212 19889198 10.1186/bcr2413 PMC2790857

[bib16] Dowsett M, Cuzick J, Ingle J, Coates A, Forbes J, Bliss J, Buyse M, Baum M, Buzdar A, Colleoni M, Coombes C, Snowdon C, Gnant M, Jakesz R, Kaufmann M, Boccardo F, Godwin J, Davies C, Peto R (2010) Meta-analysis of breast cancer outcomes in adjuvant trials of aromatase inhibitors *vs* tamoxifen. J Clin Oncol 28: 509–518 19949017 10.1200/JCO.2009.23.1274

[bib17] Dunn BK, Greene MH, Kelley JM, Costantino JP, Clifford RJ, Hu Y, Tang G, Kazerouni N, Rosenberg PS, Meerzaman DM, Buetow KH (2010) Novel pathway analysis of genomic polymorphism-cancer risk interaction in the breast cancer prevention trial. Int J Mol Epidemiol Genet 1: 332–349 21152245 PMC2998292

[bib18] Early Breast Cancer trialists’ Collaborative Group (EBCTGC) (2005) Effects of chemotherapy and hormonal therapy for early breast cancer on recurrence and 15-year survival: an overview of the randomised trials. Lancet 365: 1687–1717 15894097 10.1016/S0140-6736(05)66544-0

[bib19] Fasching PA, Loehberg CR, Strissel PL, Lux MP, Bani MR, Schrauder M, Geiler S, Ringleff K, Oeser S, Weihbrecht S, Schulz-Wendtland R, Hartmann A, Beckmann MW, Strick R (2008) Single nucleotide polymorphisms of the aromatase gene (CYP19A1), HER2/neu status, and prognosis in breast cancer patients. Breast Cancer Res Treat 112: 89–98 18049890 10.1007/s10549-007-9822-2

[bib20] Gao W, Bohl CE, Dalton JT (2005) Chemistry and structural biology of androgen receptor. Chem Rev 105: 3352–3370 16159155 10.1021/cr020456u PMC2096617

[bib21] Giwercman A, Lundin KB, Eberhard J, Stahl O, Cwikiel M, Cavallin-Stahl E, Giwercman YL (2004) Linkage between androgen receptor gene CAG trinucleotide repeat length and testicular germ cell cancer histological type and clinical stage. Eur J Cancer 40: 2152–2158 15341991 10.1016/j.ejca.2004.06.004

[bib22] Gojis O, Rudraraju B, Alifrangis C, Krell J, Libalova P, Palmieri C (2010) The role of steroid receptor coactivator-3 (SRC-3) in human malignant disease. Eur J Surg Oncol 36: 224–229 19716257 10.1016/j.ejso.2009.08.002

[bib23] Goodwin PJ, Pritchard KI (2010) Obesity and hormone therapy in breast cancer: an unfinished puzzle. J Clin Oncol 28: 3405–3407 20548001 10.1200/JCO.2010.29.5113

[bib24] Hackshaw A, Roughton M, Forsyth S, Monson K, Reczko K, Sainsbury R, Baum M (2011) Long-term benefits of 5 years of tamoxifen: 10-year follow-up of a large randomized trial in women at least 50 years of age with early breast cancer. J Clin Oncol 29: 1657–1663 21422412 10.1200/JCO.2010.32.2933

[bib25] Hanley K, Wang J, Bourne P, Yang Q, Gao AC, Lyman G, Tang P (2008) Lack of expression of androgen receptor may play a critical role in transformation from *in situ* to invasive basal subtype of high-grade ductal carcinoma of the breast. Hum Pathol 39: 386–392 18187183 10.1016/j.humpath.2007.07.007

[bib26] Hietala M, Henningson M, Törngren T, Olsson H, Jernström H (2011) Androgen receptor htSNPs in relation to androgen levels and OC use in young women from high-risk breast cancer families. Mol Genet Metab 102: 82–90 20947401 10.1016/j.ymgme.2010.09.006

[bib27] Higgins MJ, Stearns V (2011) Pharmacogenetics of endocrine therapy for breast cancer. Annu Rev Med 62: 281–293 21226615 10.1146/annurev-med-070909-182545

[bib28] Hu R, Dawood S, Holmes MD, Collins LC, Schnitt SJ, Cole K, Marotti JD, Hankinson SE, Colditz GA, Tamimi RM (2011) Androgen receptor expression and breast cancer survival in postmenopausal women. Clin Cancer Res 17: 1867–1874 21325075 10.1158/1078-0432.CCR-10-2021 PMC3076683

[bib29] Lash TL, Lien EA, Sorensen HT, Hamilton-Dutoit S (2009) Genotype-guided tamoxifen therapy: time to pause for reflection? Lancet Oncol 10: 825–833 19647203 10.1016/S1470-2045(09)70030-0 PMC2895727

[bib30] Lindstrom S, Wiklund F, Adami HO, Balter KA, Adolfsson J, Gronberg H (2006) Germ-line genetic variation in the key androgen-regulating genes androgen receptor, cytochrome P450, and steroid-5-alpha-reductase type 2 is important for prostate cancer development. Cancer Res 66: 11077–11083 17108148 10.1158/0008-5472.CAN-06-3024

[bib31] Miller WR (2006) Aromatase and the breast: regulation and clinical aspects. Maturitas 54: 335–341 16730141 10.1016/j.maturitas.2006.04.020

[bib32] Panet-Raymond V, Gottlieb B, Beitel LK, Pinsky L, Trifiro MA (2000) Interactions between androgen and estrogen receptors and the effects on their transactivational properties. Mol Cell Endocrinol 167: 139–150 11000528 10.1016/s0303-7207(00)00279-3

[bib33] Park S, Koo J, Park HS, Kim JH, Choi SY, Lee JH, Park BW, Lee KS (2010) Expression of androgen receptors in primary breast cancer. Ann Oncol 21: 488–492 19887463 10.1093/annonc/mdp510

[bib34] Peters AA, Buchanan G, Ricciardelli C, Bianco-Miotto T, Centenera MM, Harris JM, Jindal S, Segara D, Jia L, Moore NL, Henshall SM, Birrell SN, Coetzee GA, Sutherland RL, Butler LM, Tilley WD (2009) Androgen receptor inhibits estrogen receptor-alpha activity and is prognostic in breast cancer. Cancer Res 69: 6131–6140 19638585 10.1158/0008-5472.CAN-09-0452

[bib35] Pfeiler G, Konigsberg R, Fesl C, Mlineritsch B, Stoeger H, Singer CF, Postlberger S, Steger GG, Seifert M, Dubsky P, Taucher S, Samonigg H, Bjelic-Radisic V, Greil R, Marth C, Gnant M (2011) Impact of body mass index on the efficacy of endocrine therapy in premenopausal patients with breast cancer: an analysis of the prospective ABCSG-12 trial. J Clin Oncol 29: 2653–2659 21555684 10.1200/JCO.2010.33.2585

[bib36] Piersma D, Themmen AP, Look MP, Klijn JG, Foekens JA, Uitterlinden AG, Pols HA, Berns EM (2007) GnRH and LHR gene variants predict adverse outcome in premenopausal breast cancer patients. Breast Cancer Res 9: R51 17692113 10.1186/bcr1756 PMC2206727

[bib37] Punglia RS, Burstein HJ, Winer EP, Weeks JC (2008) Pharmacogenomic variation of CYP2D6 and the choice of optimal adjuvant endocrine therapy for postmenopausal breast cancer: a modeling analysis. J Natl Cancer Inst 100: 642–648 18445827 10.1093/jnci/djn100 PMC2864146

[bib38] Qiao Q, Nyamdorj R (2010) The optimal cutoff values and their performance of waist circumference and waist-to-hip ratio for diagnosing type II diabetes. Eur J Clin Nutr 64: 23–29 19690576 10.1038/ejcn.2009.92

[bib39] Ringberg A, Bågeman E, Rose C, Ingvar C, Jernström H (2006) Of cup and bra size: reply to a prospective study of breast size and premenopausal breast cancer incidence. Int J Cancer 119: 2242–2243; author reply 2244 16841335 10.1002/ijc.22104

[bib40] Rodriguez-Gonzalez G, Ramirez-Moreno R, Perez P, Bilbao C, Lopez-Rios L, Diaz-Chico JC, Lara PC, Serra-Majem L, Chirino R, Diaz-Chico BN (2009) The GGN and CAG repeat polymorphisms in the exon-1 of the androgen receptor gene are, respectively, associated with insulin resistance in men and with dyslipidemia in women. J Steroid Biochem Mol Biol 113: 202–208 19159685 10.1016/j.jsbmb.2008.12.009

[bib41] Sissung TM, Danesi R, Kirkland CT, Baum CE, Ockers SB, Stein EV, Venzon D, Price DK, Figg WD (2011) Estrogen receptor alpha and aromatase polymorphisms affect risk, prognosis, and therapeutic outcome in men with castration-resistant prostate cancer treated with docetaxel-based therapy. J Clin Endocrinol Metab 96: E368–E372 21106711 10.1210/jc.2010-2070 PMC3048329

[bib42] Somboonporn W, Davis SR (2004) Postmenopausal testosterone therapy and breast cancer risk. Maturitas 49: 267–275 15531122 10.1016/j.maturitas.2004.06.020

[bib43] Yamashita H, Takahashi S, Ito Y, Yamashita T, Ando Y, Toyama T, Sugiura H, Yoshimoto N, Kobayashi S, Fujii Y, Iwase H (2009) Predictors of response to exemestane as primary endocrine therapy in estrogen receptor-positive breast cancer. Cancer Sci 100: 2028–2033 19659610 10.1111/j.1349-7006.2009.01274.x PMC11158316

[bib44] Zhou XE, Suino-Powell KM, Li J, He Y, Mackeigan JP, Melcher K, Yong EL, Xu HE (2010) Identification of SRC3/AIB1 as a preferred coactivator for hormone-activated androgen receptor. J Biol Chem 285: 9161–9171 20086010 10.1074/jbc.M109.085779 PMC2838335

